# I was like sh*t this is gonna hurt’: Implementing Self-Sampling of Dried Blood Spots to Measure HIV Viral Load

**DOI:** 10.21203/rs.3.rs-4132978/v1

**Published:** 2024-04-02

**Authors:** Jennifer V. Chavez, Leah Davis Ewart, Ozair Ilyas, Delaram Ghanooni, José E. Diaz, Lindsay Atkins, Richard Ramos, Adriana Hernandez Garayua, Alex Stewart, Keith J. Horvath, Sabina Hirshfield, Adam W. Carrico

**Affiliations:** Florida International University; Florida International University; University of Miami; Florida International University; State University of New York; San Diego State University; University of Miami; University of Miami; University of Miami; San Diego State University; State University of New York; Florida International University

**Keywords:** acceptability, HIV viral load, methamphetamine, self-sampling, stimulants

## Abstract

**Background:**

Sexual minority men (SMM) with HIV who use stimulants may experience greater difficulties with antiretroviral therapy adherence which amplifies risk for unsuppressed HIV viral load (VL). Remote monitoring of VL could support efforts to rapidly respond to sub-optimal adherence.

**Methods:**

This qualitative study enrolled 24 SMM with HIV who use stimulants to examine experiences with two different dried blood spots (DBS) self-sampling devices (i.e., Tasso-M20 vs. HemaSpot HD) to measure VL. Participants were asked to complete self-sampling of DBS using both devices, and then participated in a 45-minute semi-structured interview. Interviews focused on ease of use, device preference, experiences with receiving and mailing kits, and barriers to participating in research. A thematic analysis was conducted to analyze interviews transcripts.

**Results:**

Twenty-two participants (92%) returned the Tasso-M20 and 21 (88%) returned the Hemaspot HD devices. Among the 22 participants that completed qualitative interviews, twenty-three codes were identified and collapsed within seven themes. Preferences for devices were based on convenience, pain and prior experiences with finger-pricking technology. Participants emphasized that clearer instructions with contingency plans for self-sampling of DBS would improve the user experience with self-sampling of DBS. Intersectional stigma (e.g., HIV, sexual minority status, and substance use) was noted as an important consideration in implementing self-sampling of DBS. Promoting decision making, or the option to choose sampling method based on personal preferences, may improve engagement and likelihood of DBS completion.

**Conclusions:**

Findings will guide the broader implementation of self-sampling of DBS to optimize VL monitoring in SMM with HIV who use stimulants.

## Background

Routine HIV viral load (VL) testing is recommended to assess the success of anti-retroviral therapy (ART) and identify individuals who may be experiencing difficulties with adherence to optimize rates of viral suppression (1). The U.S. Department of Health and Human Services (HHS) Panel on Antiretroviral Guidelines for Adults and Adolescents recommends routine VL monitoring every three to six months after initiating ART. For those with optimal ART adherence and consistently suppressed VL for more than one year, VL monitoring is recommended every six months (2).

Traditional approaches to measure VL require an in-person venipuncture to collect a peripheral venous blood sample, which represents an key barrier to regular monitoring in several marginalized, underserved populations, including people who use substances (3). People with HIV who use stimulants (i.e., methamphetamine, powder cocaine, or crack-cocaine) face multiple, intertwining barriers to engagement in care, including financial hardship, stigma, medical mistrust, homelessness, and mental health comorbidities (3–7). Self-sampling devices for dried blood spots (DBS) are feasible in this population such that participants describe this approach as convenient because it eliminates the need for in-person venipuncture (8, 9). In fact, self-sampling of DBS for laboratory-based VL testing is a medically effective measure to reduce AIDS-related mortality in Africa, and has contributed to the surveillance of HIV drug resistance (10–12). Although it has the potential to optimize HIV treatment as prevention, further research is needed to optimize procedures for implementing self-sampling of DBS in high priority populations with HIV such as people who use stimulants.

Among sexual minority men (SMM) with HIV who use stimulants, self-sampling of DBS for laboratory-based VL testing is feasible and acceptable(8, 9). However, challenges remain that are relevant to guiding the implementation of robust self-sampling procedures. In a recent study that enrolled 766 SMM with HIV using the HemSpot HF device, 61% returned a dried blood spot of sufficient volume to complete laboratory based VL testing. Notably, nearly one-in-five (i.e., 18%) of DBS specimens that were received were not of sufficient volume. Even when compared to SMM with HIV who use other substances, those who use stimulants were significantly less likely to return viable DBS (9, 13). Clearly, expanded efforts are needed to better understand the experiences of SMM with HIV who use stimulants in collecting DBS to guide more robust implementation of self-sampling for VL monitoring in this high priority population.

Solutions to some of these challenges may be identified using qualitative research methods to provide insight about how best to optimize the implementation of new approaches to improve the health of marginalized, underserved populations (14, 15). This qualitative study sought to characterize experiences of SMM with HIV who use stimulants in self-collecting DBS samples using the HemaSpot HD (Spot on Sciences) and the Tasso-M20 (Tasso, Inc.) devices. Both self-sampling devices offer a convenient alternative to in-person venipuncture for VL testing (16, 17) using different methods. We examined the experiences of 22 SMM with HIV who use stimulants in self-sampling DBS with these two devices.

## Methods

Twenty-four participants completed informed consent and were asked to conduct usability testing of two self-sampling DBS devices, Tasso-M20 and HemaSpot-HD. Twenty-two (n = 22) participants returned DBS specimens. Of the 22 participants who returned DBS specimens, 21 returned DBS specimens using both devices while one participant returned a specimen using only the Tasso-M20 device. Regardless of whether devices were returned, all 24 participants were invited to participate in a 45-minute, semi-structured, Zoom-based qualitative interview to better understand their experiences, acceptability, and preferred self-sampling device. Of the 24 consented participants, only the 22 that returned devices completed qualitative interviews. At this stage in the research, acceptability for each device is defined as the return rate, the ease expressed by participants regarding self-sampling of DBS as well as overall experience with each device.

### Participants & Recruitment

A convenience sample of SMM with HIV who use stimulants were recruited via email using an existing consent-to-contact database of individuals interested in potential research participation. Recruitment included up to three emails, sent between March and June 2022, which detailed the study purpose and provided a link to the online study screener. Interested individuals who clicked on the provided link were directed to a brief screener consent page before reaching the screener survey. Eligible individuals were a) cisgender man, b) aged 18 or older, c) comfortable reading and speaking English, d) US residency, e) reported ever having anal sex with a man, f) self-reported HIV diagnosis at least 90 days prior to screening, g) an active ART prescription AND < 90% past-month adherence on the 3-item Wilson measure in the past 30 days (18), h) screened positive for a moderate or severe stimulant use disorder (i.e., cocaine or methamphetamine) in the past 3 months with an abbreviated version of the National Institute on Drug Abuse (NIDA)-modified Alcohol, Smoking and Substance Involvement Screening Test (ASSIST) (19), and i) ownership of an iPhone or Android smartphone. Individuals who met the eligibility criteria were contacted by study staff to schedule a Zoom assessment, where they received information about the study, completed informed consent, and provided contact information so that self-sampling DBS collection devices could be mailed to their homes.

### Measures

A 9-item online screening survey was administered to assess the inclusion criteria. The survey assessed age, race/ethnicity, gender identity, comfort level with reading and speaking English, current residence, recent alcohol and other substance use (Alcohol, Smoking and Substance Involvement Screening Test [ASSIST]–8 items) (19), and self-reported ART adherence (18).

### Procedures

This study was approved by the Institutional Review Board (IRB) of [masked] and informed consent was obtained via Zoom for Healthcare prior to data collection. Semi-structured interviews were conducted by three study team members (D.G. [female], L.A. [female] and J.D. [male]) who have experience working with individuals from similar populations and had participated in qualitative interviews previously. Participants had no previous engagement with the interviewers or the study. All three study team members used an interview guide that was developed by the principal investigators (A.C., S.H., and K.H.) in consultation with the study team. The interview guide included questions on device receipt (e.g., *When your HemaSpot/Purple kit arrived, please tell me about its condition. For example, was the package intact, opened/tampered with, or damaged?), instructions (What, if any, additional information do you think would have helped you to collect your blood sample when using the device?), experiences with collecting blood samples using the devices (What was it like to prick your finger to collect blood?), shipping/returning the kits (How comfortable did you feel receiving blood collection kits at the address you provided?), the appropriateness of compensation (Do you think this was an adequate amount to compensate you for completing and returning the device?),* privacy (*How important or unimportant was privacy regarding collecting the blood sample? Why?*), social and personal barriers to research (*Thinking about your friends or other people like you, what would make it difficult for them to complete all of the steps in this study?*) as well as overall experience with each of the devices (*Overall, how would you describe your experience using the Hemaspot HD and Tasso-M20 devices?*). All interviews were conducted and recorded using the Zoom for Healthcare platform, and transcribed verbatim in English by a professional transcription company. A member of the study team (D.G.) verified the quality of the transcriptions. Participants were compensated $50 after the initial assessment visit, $50 for the follow up visit, and $30 after returning their self-sampling DBS device(s) via Amazon gift cards or secure cash applications.

### Devices for Self-Sampling of DBS and Shipping

#### Tasso-M20.

The Tasso-M20 device is a blood lancet device designed to collect whole blood for dried blood samples without the need for venipuncture(17). When participants are ready to self-collect, the Tasso device is attached to the upper arm, where a sterile lancet punctures the skin and allows blood to flow into a pod. When the pods are full (70 uL), the device is detached from the arm, and can be mailed to the receiving laboratory. The Tasso-M20 device kit includes instructions for use (written and video), the Tasso device, an alcohol wipe, a bandage, and a return bag. Figure I contains the written instructions and video instruction links for the Tasso M-20 device.

#### HemaSpot HD.

The HemaSpot HD device is another type of DBS collection device, which is designed for blood volumes of up to 160 uL (i.e., twice the volume of Tasso-M20) (16). To use the HemaSpot HD device, participants are asked to use a lancet to prick a finger, and gently apply pressurefinear the puncture site to obtain approximately 5 drops of blood. Blood drops are then applied to the center of the application surface to permeate the collection membrane and saturate the paper inside. The cartridge lid is closed and the HemaSpot HD before shipping. The Hemapsot HD kit included instructions for use (written and video), the Hemapsot HD device, an alcohol wipe, cotton pad, two lancets, a bandage, and a return bag. Figure II contains the written instructions and video instruction links for the HemaSpot HD device.

#### Shipping.

Both devices were sent in an unmarked bubble mailer, with no indication of what was included inside to protect participant confidentiality. The bubble mailer contained each of the devices with relevant collection materials (i.e., alcohol pads, cotton pads, and lancets), as well as a pre-paid bubble mailer for participants to send back their device(s) via the US Postal Service (using www.stamps.com).

### Analysis

Thematic analysis (coding reliability) was used to analyze qualitative data collected through the interviews (20). Thematic analysis is “a method for analyzing data that entails searching across a data set to identify, analyze, and report repeated patterns” (21). Given its flexibility and applicability to a wide range of research questions and study designs, thematic analysis was deemed an appropriate method to analyze these data (22–24). Five raters (J.V.C., O.I., R.R., A.C., A.H.G.) read through a subset of transcripts and developed an initial codebook. The same raters then independently coded three transcripts and met to discuss coding and refine the codebook. Feedback on the codebook was provided by an independent qualitative methodologist (S.S.), who trained all five raters as part of a graduate course in qualitative methods. The final codebook, which included 23 codes, was applied to all 22 transcripts. Percentage agreement was 89% based on a subset of three randomly selected interviews coded by two raters independently (25). Interviews were uploaded to Dedoose to facilitate collaborative coding and allow for the analysis of themes. Saturation was achieved after coding four transcripts, given that no new codes emerged during subsequent transcript analysis. The Consolidated Criteria for Reporting Qualitative Research (CERQ) guided the reporting of this study (26).

## Results

Among the 24 participants who provided informed consent, 22 who returned at least one self-sampling device for DBS, and each of these participants also completed an in-depth qualitative interview. We were unable to conduct qualitative interviews with the two participants who did not return any devices for self-sampling of DBS. Thus, the analytic sample for this qualitative investigation included the 22 participants who returned at least one self-sampling device for DBS.

As shown in Table I, the median age was 47 (IQR = 37–55) with a range of 29–70 years old. Regarding race and ethnicity, 10 were non-Hispanic White, 7 were Hispanic/Latino, 4 were Black/African American, and one was Asian/Pacific Islander. Thirteen participants reported only methamphetamine use, three reported only cocaine use, and six reported methamphetamine and cocaine co-use. ASSIST scores for individuals reporting cocaine use were in the low (n = 15) and moderate use (n = 7) categories; and those reporting methamphetamine use were in low (n = 3), moderate (n = 11) and severe (n = 8) use categories.

### Theme 1. Self-collection is feasible and acceptable.

Participants indicated that the idea of self-collection was convenient and easy to do, and that providing kits with the necessary tools (e.g., lancets, cotton pads, and pre-paid packaging) improved feasibility. They also mentioned that not having to travel to a doctor’s office also contributed to the acceptability of self-collection.

I like that idea, um, of being able to do it myself.

(Non-Hispanic African American/Black, 63 years old)

“It’s really nice to just do it at home. Easy.”

(Non-Hispanic White, 59 years old)

“It was-it would keep me from making a trip to the doctor’s office waiting and going through that, where I could just put that (Tasso-M20) in my arm and have it fill it up on its own, and just remove it when it’s filled and put it in the mail, and you’re done.”

(Non-Hispanic White, 56 years old)

### Theme 2. The device showdown: Convenience and pain are major factors involved in a preference for Tasso-M20 versus HemaSpot HD.

Participants indicated clear device preferences, which were mostly based on convenience and pain level. Some participants reported that they preferred the Tasso-M20 because it had fewer parts and required less of a “clean-up.” On the other hand, other participants reported preference for the HemaSpot HD because of their familiarity with finger pricking.

About Tasso-M20:

“It is. Are you kidding? I’m like, no, rub your arm, and you know, a little warm. Stick it in, and then watch the blood go. Oh, like, that God. You can do that—you can do that in the morning with your cup of coffee, okay [laughter].”

(Non-Hispanic African American/Black, 63 years old)

“Um, it was really interesting. And it was actually—I would prefer using that kind of kit for blood collection in the-in the future as well, um, ‘cause it was just—it was so easy. And it wasn’t—it-it—anything that I expected.”

(Non-Hispanic White, 29 years old)

“Um, like I said, it’s easy to clean up. There’s no—there’s not too many factors when it come[s] to it. And I don’t have any hazardous waste afterwards. Basically, everything that’s hazardous is going with the package.”

(Hispanic/Latino 33 years old)

“I like that one on the arm. The arm one was super easy, super, super fun. I mean, I shouldn’t say fun.”

(Non-Hispanic Asian, 39 years old)

About HemaSpot HD:

“It was—um, it was easy. I was surprised at, like, how much my finger bled. [Laughter] Um, so I had to make sure that—like, in the beginning, I had to wipe off the excess blood and then just drop it—to make sure it got into the kit, the-the l—the, um, receptacle like it was—’cause it’s kinda small…”

(Non-Hispanic White, 52 years old)

“But it was—um, other than that, it was-it was, um, it was-it was very—it was very easy. I was, um—I-I—I don’t know, I guess I expected that for, um, for something that was, you know, self-administered, there’d be a bit more difficulty to the process, but it was very easy.”

(Hispanic/Latino, 39 years old)

“It was a breeze. And it was really—as long as I could trust that there’s enough—um, depending on what labs I needed done, you know, every six months or whatever— as long as I could trust that that’s— whatever I can give in that little, you know, kit, if—that I can trust that that’s gonna be enough [for an HIV viral load], I feel con dent ‘cause it’s—it was so easy.”

(Non-Hispanic White, 59 years old)

“Um, it-it—I was like—like I said, so I was a little put back at first, and I was like, “Sh*t, this is gonna hurt.” And I was just like, “Let me just get it over with.” One, two, three, and click. Once it made the click sound, it was like, “Okay, it’s not that bad.” It really—- really wasn’t that bad.”

(Hispanic/Latino, 32 years old)

### Theme 3. Clear instructions are crucial to self-sampling of DBS.

Participants described how they relied heavily on both written and video instructions. Some expressed how their understanding of the written instructions was complemented by the video. Participants’ recommendations included adding more realistic images to written instructions (e.g., images of actual people conducting the blood collection), not relying on illustrations, and including contingency plans.

“It seemed like that based on the instructions that I read, and in the process once I did it, it-it felt—like I felt comfortable that-that I had achieved what I needed to do with it, you know. Like I-I was successful like I felt like.”

(Non-Hispanic White, 52 years old)

“What do I do if I can’t make this work?” Um, you know. And, uh—yeah. So that was a big problem I ran into with that one. And considering it’s happened to me twice now or two other—two times, it’s—I—it—I mean, it may be something about me or whatever, but, um, it doesn’t seem to be working too well.”

(Non-Hispanic White, 50 years old)

“Um, the illustrations were not real pictures. They were—you know, the simulated picture. But not—I think it had definitely been ill—it would have been-it would have been better to see it, um, see a real picture.”

(Non-Hispanic Black/African American, 37 years old)

About the Tasso-M20 Instructional Video:

“Yes. Um, the video was definitely a lot more helpful, especially for the one that went on the shoulder.”

(Non-Hispanic White, 29 years old)

About the HemaSpot HD Instructional Video:

“And that was-that was a great thing to have as a QR code. You just scan it there. The video was very well informative—oh. Well, yes. The video was informative.”

(Non-Hispanic Black/African American, 37 years old)

### Theme 4. Privacy and confidentiality are important considerations.

Participants described how the discreet packaging protected confidentiality. They also reported that privacy would be more important if they did not live in an “accepting” neighborhood or lived with family. Participants enjoyed the additional privacy able to self-collect their blood samples at home.

“I don’t share my business with anybody el—anybody else, so, I mean, it’s kind of a—I liked the fact that it was sent to the-to the house, and, you know, it was—you know, nobody really had to be involved with it or whatever.”

(Non-Hispanic White, 44 years old)

“I mean, privacy is really important to me. Um, I just—I guess I have confidence in the fact that it’s a university study and, um, you know, we’re—uh, the—each person is rec—um, is assigned a certain, um, number code. Um, I-I feel that my data is safe.”

(Non-Hispanic White, 45 years old)

“So, if I’m not living in a place where I can be my free self, then sure, there might be some-some hesitation there.”

(Non-Hispanic Asian, 39 years old)

“Well, I mean, I guess somebody who doesn’t have the privacy in their own home to be able to do something like that, if-if they’re living with other people that-that they haven’t told that they are HIV-positive, then they might have a problem being able to get away, or go into the bathroom, or if they have their own bedroom, to go in there to do it. Um, and likewise with the stigma. They wouldn’t want somebody to see them doing it, and then that person ask questions. “Well, why or what are you doing?” “Why are you doing this?” “What’s that for?” Um, I can see where those two might be problems.”

(Non-Hispanic White, 56 years old)

### Theme 5. Despite the ease for some individuals, barriers to research persisted for others.

Although there were several aspects of the study that made participation easier, participants discussed how people with substance use disorders and individuals with disabilities may struggle to complete all aspects of the study.

“Yeah, I was gonna say, like, the primary thing that I can think of would be the drug use that, um, perhaps they’re in a bad place and, even though they seemed, like, at the time committed to doing this, they maybe didn’t have follow through because they were using too much, um, which can happen. Um, um, and then the other barrier would be the—depending upon what type of residency they have, um, if it’s-if it’s not their own home, there might be some apprehension to receiving the package, um, especially if it’s like a-a room shared kind of situation.”

(Non-Hispanic White, 33 years old)

“So it’s easy for me. And I’m not disabled. I can move around easily. So, um, it wasn’t a problem. But I think just maybe in some—uh, some people with HIV long term may—you know, maybe people that are older and disabled and might have a little trouble just, you know, getting to the post office or whatever.”

(Hispanic White, 59 years old)

Participants also identified trust in research and intersectional stigma (e.g., HIV, substance use, sexual minority status) as barriers to participation, but also mentioned the importance of structural barriers, like housing.

“More like a stigma or just, you know, their-their own personal—just, you know, feeling some type of way because of the situation. Um, anything-anything that has to do with substance abuse, they’re very, like, private or anything like that.”

(Hispanic/Latino, 33 years old)

“Um, probably I would say, um, housing maybe. Some place people can do it, you know, confidentially. And then maybe mistrust in the medical system, especially those that may be from African-American descent.”

(Non-Hispanic White, 51 years old)

“Um, just if people—I think stigma would be part of, you know, having a housing, so people don’t see you doing it, and asking you questions. Then you may have to disclose, you know, why you’re doing this, or you have to lie, you know. So, I think the stigma is still there regarding, um, HIV. So, I think that’s definitely probably one of ‘em. That’s why I was saying like if somebody had their own place to do it, then they don’t have to worry so much about that.”

(Non-Hispanic White, 51 years old)

### Theme 6. Compensation was generally considered appropriate.

Participants generally indicated that compensation was proportional to the time burden and pain experienced. Participants recommended including other methods of payment (e.g., gift cards other than Amazon and other cash applications).

“Just ‘cause, I mean, it really wasn’t a whole lotta time, and, um, uh—- you know, it’s a hundred—what is it? —$130. That’s not bad for a couple hours’ work.”

(Non-Hispanic White, 44 years old)

“It would’ve been nice to have more, but, you know, it-it is still something that, you know, that is gonna—that is much appreciated.”

(Non-Hispanic White, 50 years old)

“I mean, um, es-especially now that, you know, gas has gone up since then, um, I would probably say maybe like—I don’t know. Um, 40, 50 max. I mean, I still think 30 would still be adequate enough now. It’s not like, um, it was that difficult or, you know, I had to take a sick day or something,”

(Hispanic/Latino, 29 years old)

“The original sur—yeah, sur—the original survey, I would do the 30. And then the actual process [self-collection], I would do the 50, just because I feel like the compensation goes more with the process, like they match up more that way.”

(Hispanic/Latino, 22 years old)

Some participants indicated that compensation amounts should be reallocated to different study tasks (e.g., $30 for survey completion versus $50 for device return).

“I mean, technically speaking the two phone calls [assessment and follow-up] are longer than the amount of time it should’ve taken to collect the blood. But I just felt like that was so much more invasive.”

(Non-Hispanic White, 52 years old)

### Theme 7. Previous experience with self-sampling contributed to acceptability.

Participants reported that previous experience or exposure to finger pricking, or any type of self-collected blood sample, made collecting blood samples using the devices less challenging. Participants explained how previous experience with self-sampling for other chronic medical conditions (e.g., diabetes) made the study procedures less daunting and provided some level of “peace of mind.”

“Back to my previous experience with, uh—in the—with work with the diabetes field, um, there was a-a device, uh, called the Omnipod that was like a big—it’s a very similar type situation, but, uh, it actually delivered insulin. So, but-but it’s gonna get, uh, kind of prick the-the skin, if I remember right, so I was familiar with the concept of how that would work.”

(Non-Hispanic White, 52 years old)

“Um, so I do have a father that’s diabetic, so have dealt with pricking the finger, you know, like that whole process. I have had—I’ve dealt with that. I myself am pre-diabetic, so I’ve—I’ve had to, you know, test my blood or whatever in that aspect. So, I was comfortable with it because I’ve-I’ve already done it before.”

(Hispanic/Latino, 33 years old)

“So, I-I had to use the second lancet to-to-to do it on the finger. Um, but definitely, um, my past experience with that was kinda helpful.”

(Non-Hispanic White, 29 years old)

## Discussion

This qualitative study is among the first to assess usability testing and preferences between two self-sampling DBS kits. We examined experiences of SMM with HIV who use stimulants in self-sampling of DBS using two commercially available devices. Better understanding the experiences of this high priority populations with self-sampling of DBS is critical to optimizing usability and broader implementation of novel approaches to VL monitoring (27). Our findings underscore the importance of having more than one choice for devices in enhancing acceptability. Some participants expressed clear preferences for the Tasso-M20 or HemaSpot HD based primarily upon convenience, pain level, ease of use, prior experience with finger pricking, and the presence of clear instructions. Expanded efforts are needed to develop and validate multiple self-sampling devices for remote VL measurement.

Intersectional stigma related to HIV, sexual minority status, and stimulant use emerged as an important consideration in the implementation of self-sampling of DBS for remote VL monitoring. Enacted and anticipated intersectional stigma have previously been described as barriers to participating in clinic-based HIV testing and viral load monitoring (28, 29), and have been associated with lower viral suppression and antiretroviral therapy adherence in PWH (30–32). Individuals reporting discriminatory or stigmatizing experiences, particularly in health care settings, are also less likely to engage in HIV care services (33), highlighting the importance of considering stigma (and preventing future stigmatization), particularly among those with multiple minoritized identities (34). At the same time, others anticipated difficulties in finding sufficient privacy to self-sample DBS in shared living arrangements where they had not disclosed their HIV status or sexual minority identity. Non-self-disclosure has been associated with increased anticipated and internalized stigma, and has its own set of poor health outcomes, including poor ART adherence, and psychological distress (35).

Choice and the consideration of personal preferences emerged as crucial factors influencing engagement for DBS self-sampling. The option to self-sample for VL monitoring, as an alternative when traditional venipuncture is not readily accessible, may improve engagement of individuals who are otherwise not adherent to VL monitoring recommendations by promoting self-efficacy and autonomy. Furthermore, research has demonstrated that when individuals are provided with a choice of screening modality, adherence rates increase, and a majority opt for self-sampling (36). Empowering SMM with HIV who use stimulants to supplement their VL monitoring using DBS self-sampling may thus help to reduce their disengagement from HIV care.

Participants also provided actionable feedback that our team has leveraged to improve usability of self-sampling kits for DBS. Written instructions were revised to include real images, and not illustrations of sample collection instructions. Also, in line with the feedback received, the compensation for device return for the parent study has doubled to ensure it is proportional to the time burden and physical discomfort. Lastly, we created an instructional sheet that answers commonly asked questions and contingency plans to ensure participants provide an adequate sample volume for VL measurement.

Findings from this study should be interpreted in context of some limitations. Although the sample size was sufficient for qualitative analyses, our estimates of device return should be consider preliminary. Larger, more representative samples are needed for quantitative studies examining rates and correlates of device return in this high priority population. Semi-structured interviews are also prone to unintentional bias, including observer and social desirability bias. One of the benefits of our general inductive approach to assessing barriers and facilitators to self-sampling of DBS was the emergence of intersectional stigma as an important consideration. Mixed methods research is needed to better characterize the role of intersectional stigma in self-sampling of DBS for remote VL monitoring in this high priority population. Future studies should continue to leverage remote procedures, including online surveys and video interviews to facilitate inclusive participation and address the potential role of intersectional stigma.

Despite these limitations, findings from this qualitative study highlight the potential promise of remote VL monitoring to optimize HIV treatment as prevention in SMM with HIV who use stimulants. Results highlight that expanded efforts are needed to provide options for different self-sampling devices as well as refine instructions (written and video) to include contingency plans. Efforts to implement self-sampling of DBS for remote VL monitoring among SMM with HIV who use stimulants should attend the importance of intersectional stigma in the lived experience of this high priority population.

## Figures and Tables

**Figure 1:**
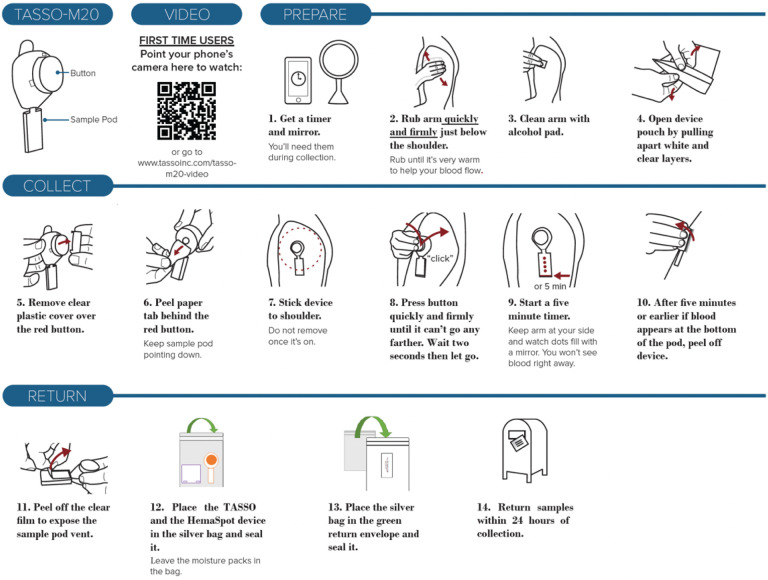
Written instructions and video instruction link for the Tasso M-20 device

**Figure 2:**
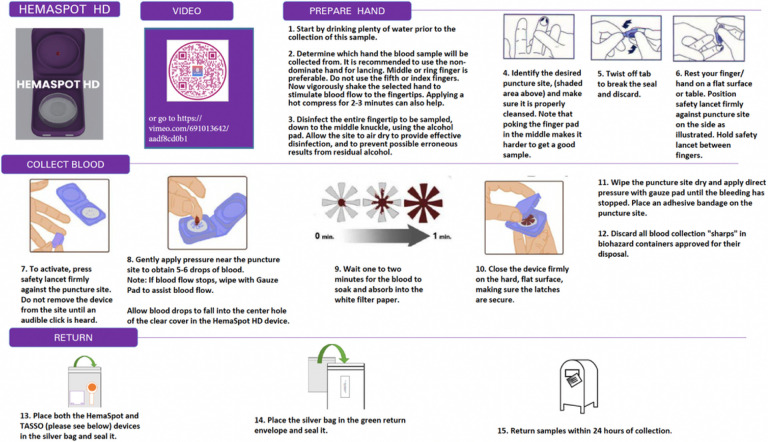
Written instructions and video instruction link for the HemaSpot HD device

**Table I.** Characteristics of enrolled participants who completed a qualitative interview (N = 22)

Median age	47 (IQR = 37–55)	
	n	%
**Race**		
White	10	45%
Black/African American	4	18%
Asian	1	5%
Hispanic/Latino White	7	32%
**Cocaine Use Severity**		
Low	15	68%
Moderate	7	32%
Severe	0	0%
**Methamphetamine Use Severity**		
Low	3	14%
Moderate	11	50%
Severe	8	36%

## Data Availability

The datasets used and/or analysed during the current study are available from the corresponding author on reasonable request.
